# Tea and liver fibrosis: a bibliometric analysis of mechanisms, translational challenges, and emerging trends

**DOI:** 10.3389/fnut.2026.1930581

**Published:** 2026-08-21

**Authors:** Fei Yu, Sihan Yin, Jiayu Zhu, Kewei Sun

**Affiliations:** 1 The First Clinical College of Chinese Medicine, Hunan University of Chinese Medicine, Changsha, Hunan, China; 2 Department of Hepatology, The First Hospital of Hunan University of Chinese Medicine, Changsha, Hunan, China

**Keywords:** bibliometric analysis, bioavailability, clinical applications, liver fibrosis, tea

## Abstract

**Objective:**

Although tea and its bioactive constituents, particularly epigallocatechin gallate (EGCG), demonstrate promising anti-liver fibrotic potential, the global research landscape has yet to be systematically characterized through quantitative visualization, leaving research priorities and clinical translational barriers unclear. This bibliometric analysis aims to delineate the current status, emerging hotspots, and developmental trends in tea-related liver fibrosis research, providing strategic insights for future studies.

**Methods:**

A dual-database search with reciprocal verification was conducted in the Web of Science Core Collection (WoSCC) and Scopus using refined search strategies. Bibliometric analyses and visualizations were performed using R (v4.5.1), VOSviewer (v1.6.19), and CiteSpace (v6.4.R1).

**Results:**

Annual publication output in this field has increased steadily. China led in publications (*n* = 120), with Nutrients and Frontiers in Pharmacology as the leading journals. Tea bioactives exert anti-fibrotic effects through: (1) molecular mechanisms, including inhibition of hepatic stellate cell activation via TGF-β/Smad, Nrf2, NF-κB, AMPK, and MAPK pathways; (2) gut-liver axis modulation, such as intestinal microbiota remodeling, bile acid homeostasis restoration, and barrier integrity enhancement; (3) synergistic effects with other natural products; and (4) epidemiological evidence showing that regular tea/caffeine intake reduces fibrosis progression, cirrhosis, and hepatocellular carcinoma risk. However, EGCG clinical translation is limited by poor solubility, environmental instability, rapid clearance, low oral bioavailability, and dose-dependent hepatotoxicity. Nano-delivery systems and structural modifications have been developed to enhance its bioavailability.

**Conclusion:**

This study elucidates the therapeutic potential of tea and its bioactive constituents against liver fibrosis, encompassing their pharmacological mechanisms, emerging molecular targets, and bioavailability enhancement strategies. With advances in nanomedicine and pharmaceutical technologies, coupled with rigorously designed multicenter randomized controlled trials, tea-derived bioactives are anticipated to overcome current limitations and establish clinical value in liver fibrosis prevention and adjuvant therapy.

## 1 Introduction

Liver fibrosis is a reversible pathological repair response triggered by persistent chronic liver injury of diverse etiologies, including metabolic dysfunction-associated steatotic liver disease (MASLD), viral hepatitis, alcoholic liver disease, and autoimmune hepatopathy (1). Uncontrolled progressive fibrosis leads to excessive deposition of extracellular matrix (ECM) driven by activated hepatic stellate cells (HSCs), eventually progressing to irreversible cirrhosis, portal hypertension and hepatocellular carcinoma (HCC) (2, 3), imposing a heavy global public health and economic burden. At present, clinical anti-fibrotic therapies mainly target primary liver diseases to delay lesion progression, yet few agents effectively reverse established fibrosis; approved targeted drugs such as Farnesoid X receptor (FXR) agonists carry notable adverse effects, and their long-term safety remains unclear (4). Therefore, exploring safe, multi-target natural dietary interventions has become a priority in hepatology research.

Tea, one of the world’s most widely consumed beverages, contains abundant bioactive components including epigallocatechin gallate (EGCG), theaflavins, theabrownins, tea polysaccharides, and caffeine. *In vitro* and *in vivo* studies have demonstrated that these phytochemicals exert hepatoprotective and anti-fibrotic effects through multiple molecular mechanisms, including suppression of oxidative stress, attenuation of inflammatory cascades, inhibition of HSC transdifferentiation, and regulation of the gut–liver axis (5, 6). Specifically, existing mechanistic studies have clarified that tea constituents modulate core signaling pathways such as Transforming Growth Factor-β (TGF-β)/Smad, Nuclear Factor-kappa B (NF-κB), Adenosine Monophosphate-Activated Protein Kinase (AMPK), and Mitogen-Activated Protein Kinase (MAPK) (7, 8), thereby interrupting the fibrogenic process.

In parallel, accumulating epidemiological evidence supports the clinical relevance of habitual tea consumption. Cohort and cross-sectional studies have shown that regular tea intake correlates with reduced risks of hepatic steatosis, significant fibrosis, cirrhosis, and liver malignant transformation (9, 10), underscoring the translational potential of tea-derived bioactives in chronic liver disease management.

Nevertheless, relevant research is scattered across nutrition, pharmacology, and gastroenterology journals, lacking a systematic, quantitative overview of the whole-field development trajectory, mainstream journals, evolving themes, and emerging frontiers. Bibliometric analysis serves as a quantitative research tool to objectively sort literature distribution, visualize cooperative networks, excavate research hotspots, and predict future trends, which can overcome the limitations of traditional narrative reviews relying on subjective literature screening (11).

## 2 Materials and methods

### 2.1 Data collection

We selected the Web of Science Core Collection (WoSCC) and Scopus databases as the primary sources for comprehensive bibliometric analysis, with data retrieved on May 20, 2026. After excluding irrelevant records, 275 eligible publications were identified from WoSCC and 391 from Scopus. Clinical trial data were additionally retrieved from PubMed. The search strategies were as follows: WoSCC:((((((((TS = (liver fibrosis)) OR TS = (hepatic fibrosis)) OR TS = (liver fibrogenesis)) OR TS = (liver cirrhosis)) OR TS = (hepatic cirrhosis)) AND TS = (tea)) AND LA = (English)) AND DT = (Article OR Review)) AND DOP = (2012-01-01/2026-05-20); Scopus: TITLE-ABS-KEY (liver fibrosis) OR TITLE-ABS-KEY (hepatic fibrosis) OR TITLE-ABS-KEY (liver fibrogenesis) OR TITLE-ABS-KEY (liver cirrhosis) OR TITLE-ABS-KEY (hepatic cirrhosis) AND TITLE-ABS-KEY (tea) AND (LIMIT-TO (DOCTYPE, “ar”) OR LIMIT-TO (DOCTYPE, “re”)) AND (LIMIT-TO (LANGUAGE, “English”)) AND PUBYEAR > 1954 AND PUBYEAR < 2025; PubMed: (((((liver fibrosis[Title/Abstract]) OR (liver fibrosis[Title/Abstract])) OR (liver fibrogenesis[Title/Abstract])) OR (liver cirrhosis[Title/Abstract])) OR (hepatic cirrhosis[Title/Abstract])) AND (tea[Title/Abstract]) AND ((“1975/01/01”[Date-Publication]: “2026/05/20”[Date-Publication])) Filters: Clinical Trial, English. An independent multi-database retrieval strategy was employed in this study: WoSCC served as the primary data source for bibliometric analysis; Scopus was used to explore research trends, hotspots, and thematic evolution, aiming to supplement and cross-validate WoSCC findings; PubMed was utilized for clinical trial retrieval and evaluation, with a focus on clinical translation evidence for tea in liver fibrosis treatment.

### 2.2 Data analysis

Bibliometric analyses and visualizations were performed using R software (version 4.5.1) with the bibliometrix package (version 5.0.1), VOSviewer (version 1.6.19), and CiteSpace (version 6.4.R1), while OriginPro 2025 was employed for temporal publication trend analysis. Journal impact factors were obtained from the 2025 Journal Citation Reports (JCR). Each database was analyzed independently. VOSviewer generated collaboration networks at the country and institutional levels, as well as document co-citation and keyword co-occurrence maps. In co-authorship analyses, a minimum threshold of two publications was applied for both countries and institutions. For co-citation analysis, only documents receiving ≥ 17 citations were retained. Keyword standardization was achieved by including terms appearing in at least three WoSCC records, with generic terms such as “tea” and “liver fibrosis” (and their variants) excluded. Emerging research fronts were identified through CiteSpace’s time-slicing functionality. By integrating these analytical tools, we delineated the research framework of tea in the treatment of liver fibrosis.

## 3 Results

### 3.1 General analysis

**FIGURE 1:**
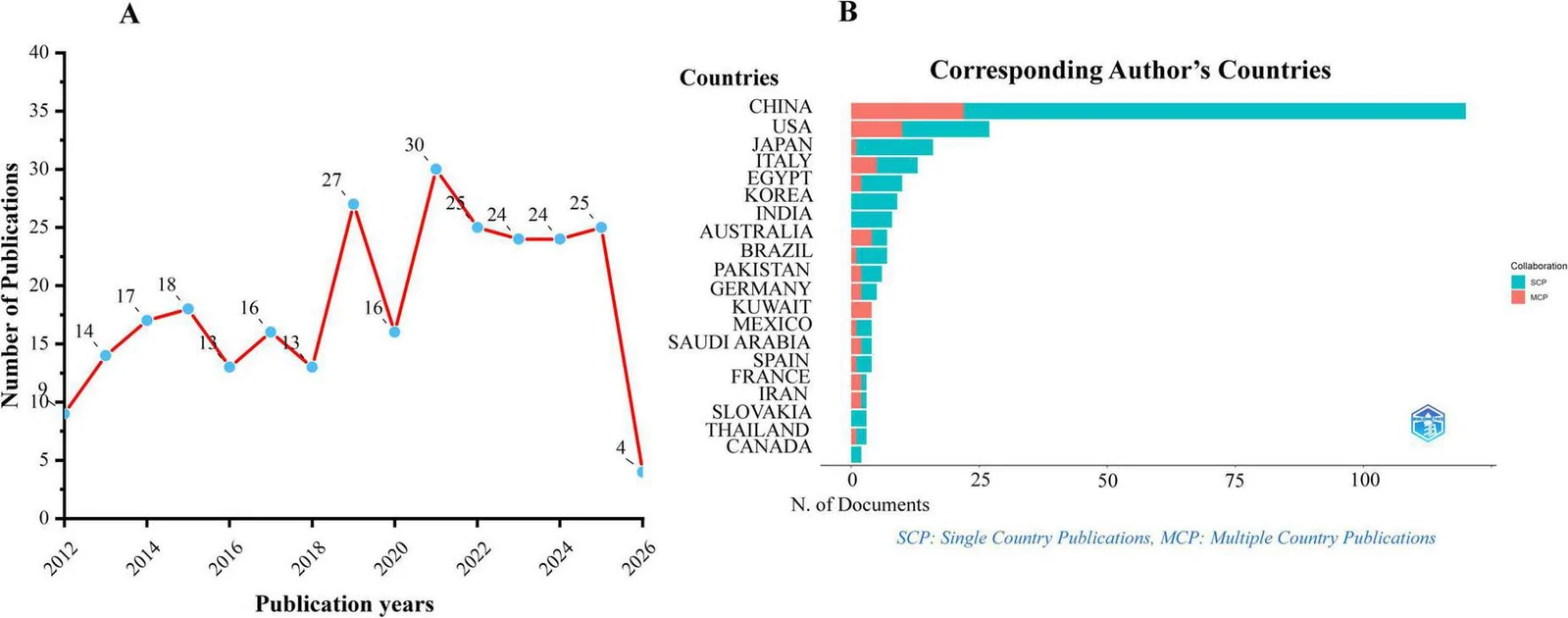
Annual publication output trends in tea for liver fibrosis **(A)**; trends of annual publication outputs **(B)**.

**FIGURE 2:**
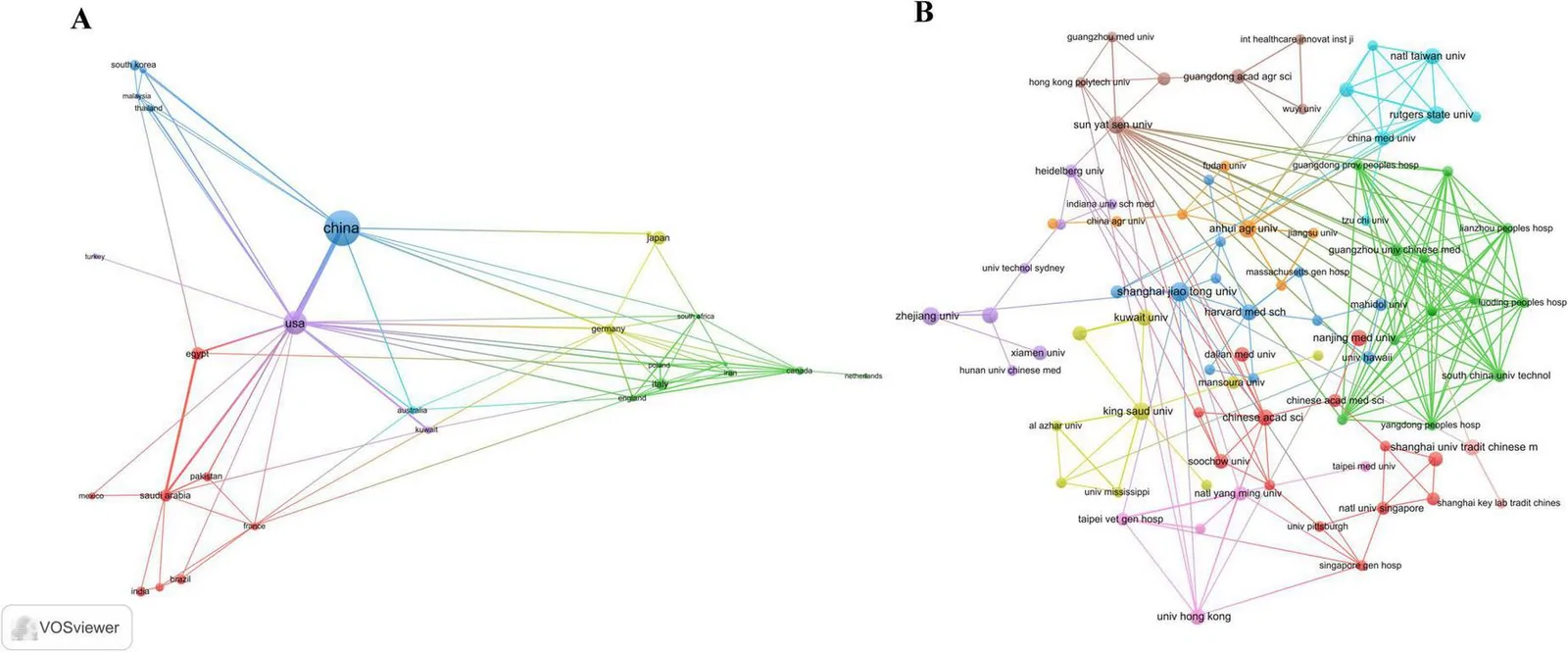
Countries/regions and institutional collaboration maps of tea for liver fibrosis. **(A)** International collaboration map. **(B)** Institutional collaboration map.

**FIGURE 3:**
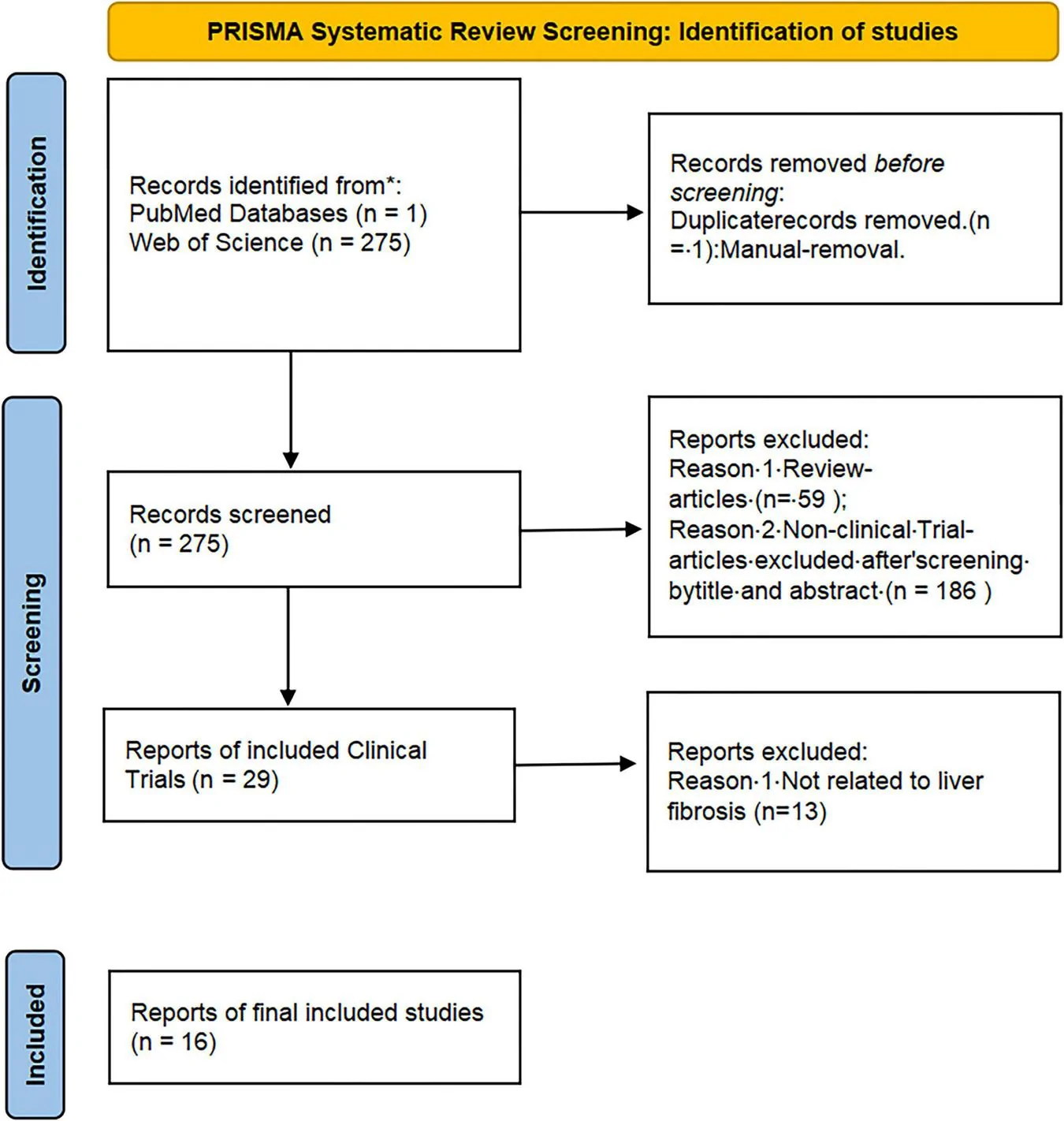
PRISMA flow diagram for the study selection process.

A total of 275 publications were retrieved from WoSCC. As shown in Figure 1A, annual publication output exhibited an overall upward trend from 2012 to 2026. Based on the affiliations of corresponding authors, CHINA (*n* = 120) contributed the largest number of publications, followed by the USA (*n* = 27), KOREA (*n* = 21), and JAPAN (*n* = 16) (Figure 1B and Table 1). Furthermore, Figure 2A demonstrates that China maintained the most extensive international collaborations in this field. The collaboration network highlighted leading institutions with notable research productivity and collaborative activities, including Shanghai Jiao Tong University (*n* = 7) (Figure 2B; detailed data are provided in Annex 1). A total of 16 clinical studies were included from PubMed and WoSCC databases following the inclusion workflow depicted in Figure 3, after duplicate removal.

**TABLE 1:** Most relevant countries by corresponding authors in tea for liver fibrosis.

Country	Articles	Articles %	SCP	MCP	MCP %
China	120	43.6	98	22	18.3
USA	27	9.8	17	10	37
Japan	16	5.8	15	1	6.3
Italy	13	4.7	8	5	38.5
Egypt	10	3.6	8	2	20
Korea	9	3.3	9	0	0
India	8	2.9	8	0	0
Australia	7	2.5	3	4	57.1
Brazil	7	2.5	6	1	14.3
Pakistan	6	2.2	4	2	33.3

MCP, multiple-country publication; SCP, single-country publication.

### 3.2 Journal analysis and visualization

Using the bibliometrix and ggplot2 packages in R software (version 4.4.1), we analyzed the journals publishing research on tea in liver fibrosis treatment. Additionally, co-citation journal analysis was performed using VOSviewer (version 1.6.19). The results showed that the 275 WoSCC-indexed articles were distributed across 157 distinct academic journals (Annex 2).

**FIGURE 4:**
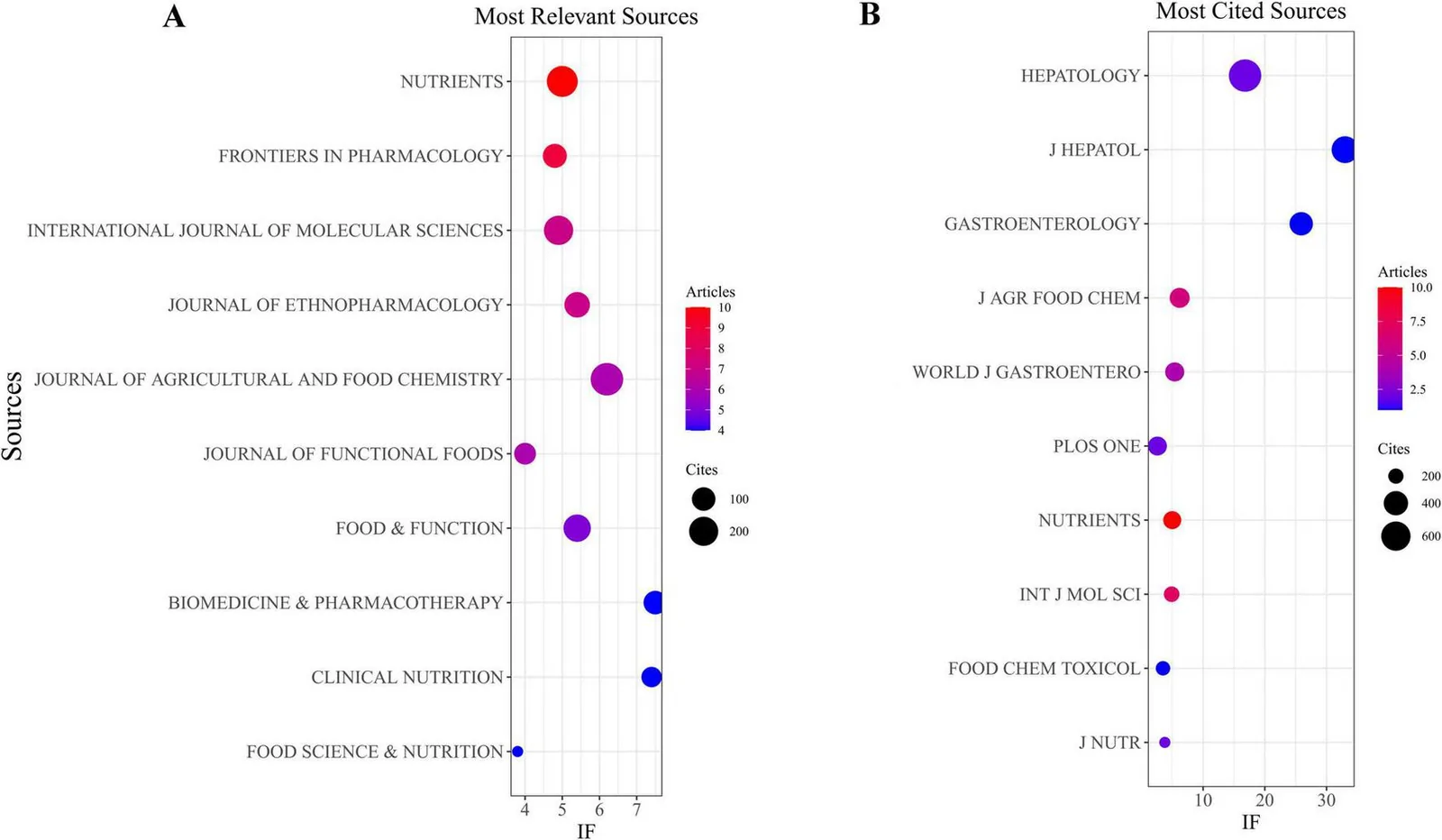
Journal with the largest number of articles published and the journal with the largest number of citations. **(A)** Journal with the largest number of articles published. **(B)** Journals with the largest number of citations.

Regarding publication sources, as shown in Table 2, the two most prolific journals were NUTRIENTS (*n* = 10, IF = 5), FRONTIERS IN PHARMACOLOGY (*n* = 9, IF = 4.8) (Figure 4A). We also examined highly cited journals; as presented in Table 3 and Figure 4B, the most frequently cited journals were HEPATOLOGY (*n* = 754, IF = 16.8), JOURNAL OF HEPATOLOGY (*n* = 494, IF = 33), GASTROENTEROLOGY (*n* = 371, IF = 25.9).

**TABLE 2:** Top 10 journals with the most published articles.

Journal	Documents	IF (2025)	Cites
Nutrients	10	5	239
Frontiers in Pharmacology	9	4.8	107
International Journal of Molecular Sciences	7	4.9	203
Journal of Ethnopharmacology	7	5.4	131
Journal of Agricultural and Food Chemistry	6	6.2	278
Journal of Functional Foods	6	4	76
Food and Function	5	5.4	164
Biomedicine and Pharmacotherapy	4	7.5	100
Clinical Nutrition	4	7.4	60
Food science and Nutrition	4	3.8	10

**TABLE 3:** Top 10 journals with the most cited journals.

Journal	Cites	IF (2025)	Documents
Hepatology	754	16.8	2
Journal of Hepatology	494	33	1
Gastroenterology	371	25.9	1
Journal of Agricultural and Food Chemistry	278	6.2	6
World Journal of gastroenterology	259	5.4	4
PLoS ONE	256	2.6	2
Nutrients	239	5	10
International Journal of Molecular Sciences	203	4.9	7
Food and Chemical Toxicology	192	3.5	1
Journal of Nutrition	178	3.8	2

**FIGURE 5:**
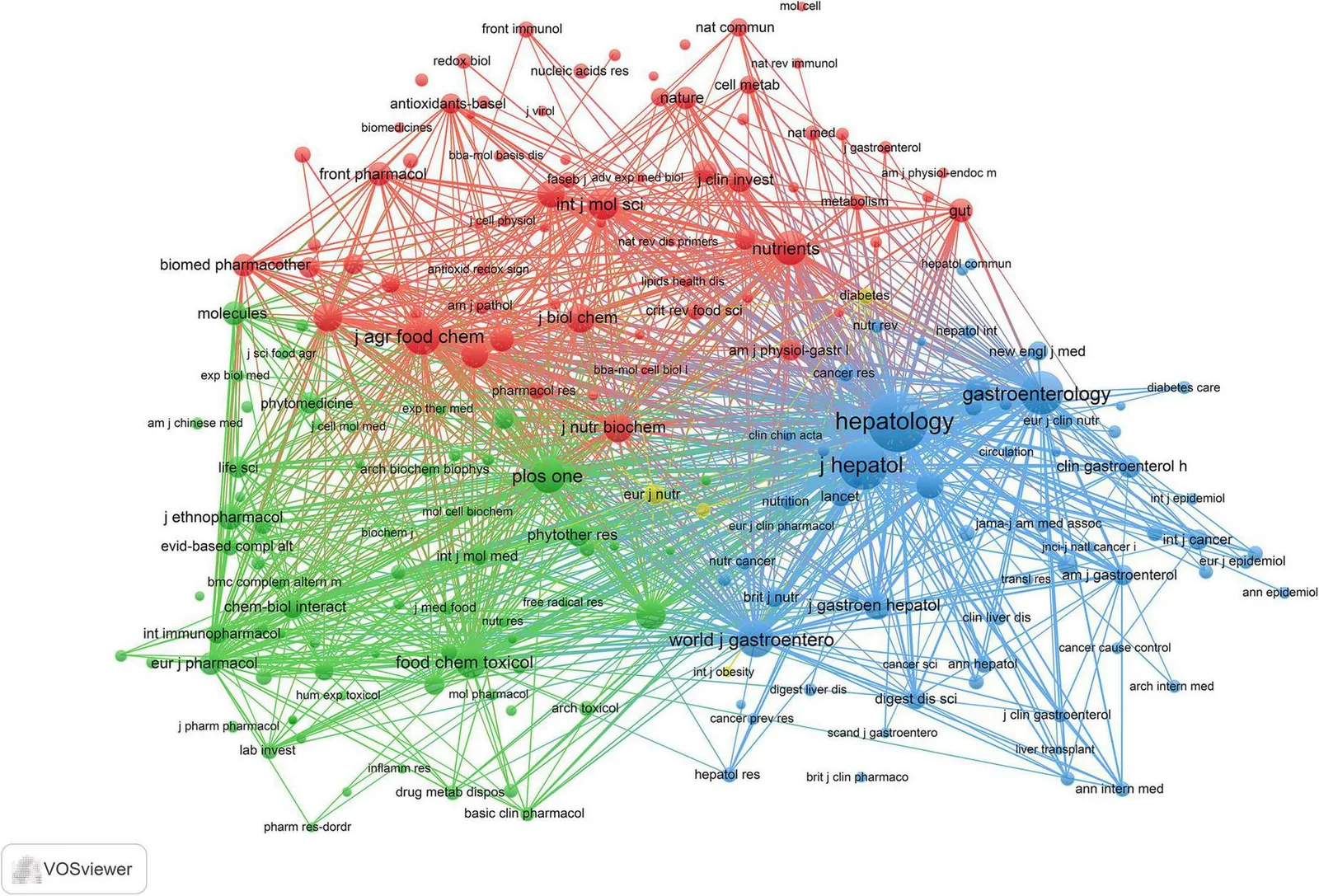
Co-cited journals involved in tea for liver fibrosis.

The co-citation journal visualization analysis (Figure 5) indicates that NUTRIENTS and FRONTIERS IN PHARMACOLOGY are highly influential journals in the field of tea for liver fibrosis. These findings demonstrate substantial research output published in high-impact journals, reflecting broad international academic recognition and underscoring the significant scholarly value of this research direction.

### 3.3 Most cited references and reference burst

Using the bibliometrix package in R software, we identified the top 20 most-cited articles in the field of tea for liver fibrosis treatment. In WoSCC, each article received ≥ 139 citations, spanning 19 distinct journals; in Scopus, each article received ≥ 192 citations, also distributed across 19 distinct journals (Table 4).

**TABLE 4:** Top 20 cited references related to tea for liver fibrosis.

WoSCC database				Scopus database			
Paper	DOI	Total citations	TC per year	Paper	DOI	Total citations	TC per year
Wang XB, 2016, Cell Metab	10.1016/j.cmet.2016.09.016	350	31.82	Leung C, 2016, Nat Rev Gastroenterol Hepatol	10.1038/nrgastro.2016.85	931	84.64
Vallianou N, 2022, Biomolecules	10.3390/biom12010056	241	48.20	Firuzi O, 2011, Curr Med Chem	10.2174/092986711803414368	415	25.94
Salomone F, 2016, Liver Int	10.1111/liv.12975	220	20.00	Kidd PM, 2009, Altern Med Rev		358	19.89
Chu CY, 2017, Biomed Res Int	10.1155/2017/5615647	189	18.90	Wang X, 2016, Cell Metab	10.1016/j.cmet.2016.09.016	351	31.91
Bravi F, 2013, Clin Gastroenterol H	10.1016/j.cgh.2013.04.039	182	13.00	Yin S-Y, 2013, Evid-Based Complement Altern Med	10.1155/2013/302426	345	24.64
Oz HS, 2013, Front Immunol	10.3389/fimmu.2013.00132	173	12.36	CIESEK S, 2011, HEPATOLOGY	10.1002/hep.24610	275	17.19
Xiao J, 2014, Eur J Nutr	10.1007/s00394-013-0516-8	172	13.23	Bunchorntavakul C, 2013, Aliment Pharmacol Ther	10.1111/apt.12109	266	19.00
Edgar JA, 2015, Chem Res Toxicol	10.1021/tx500403t	170	14.17	Borek C, 2004, Integr Cancer Ther	10.1177/1534735404270578	257	11.17
Zhang KK, 2023, ACS Nano	10.1021/acsnano.3c04449	166	41.50	Bito T, 2020, Nutrients	10.3390/nu12092524	249	35.57
Li S, 2018, Oxid Med Cell Longev	10.1155/2018/8394818	162	18.00	Posadzki P, 2013, Br J Clin Pharmacol	10.1111/j.1365-2125.2012.04350.x	246	17.57
Marcolin E, 2012, J Nutr	10.3945/jn.112.165274	153	10.20	Lecumberri E, 2013, Clin Nutr	10.1016/j.clnu.2013.03.008	243	17.36
Xiao J, 2012, J Ethnopharmacol	10.1016/j.jep.2011.11.033	153	10.20	Klatsky AL, 1992, Am J Epidemiol	10.1093/oxfordjournals.aje.a116433	241	6.89
Van De Wier B, 2017, Crit Rev Food Sci	10.1080/10408398.2014.952399	151	15.10	Salomone F, 2016, Liver Int	10.1111/liv.12975	231	21.00
Saab S, 2014, Liver Int	10.1111/liv.12304	146	11.23	Ha H-L, 2010, World J Gastroenterol	10.3748/wjg.v16.i48.6035	209	12.29
Marventano S, 2016, Clin Nutr	10.1016/j.clnu.2016.03.012	145	13.18	Bagnis CI, 2004, Am J Kidney Dis	10.1053/j.ajkd.2004.02.009	209	9.09
Zhang ZC, 2021, Trends Food Sci Tech	10.1016/j.tifs.2021.05.023	144	24.00	Afzal M, 2015, Inflammopharmacology	10.1007/s10787-015-0236-1	206	17.17
Setiawan VW, 2015, Gastroenterology	10.1053/j.gastro.2014.10.005	141	11.75	Sheikh NM, 1997, Arch Intern MED	10.1001/archinte.157.8.913	205	6.83
Fan JG, 2013, J Gastroen Hepatol	10.1111/jgh.12244	140	10.00	Xiao J, 2014, Eur J Nutr	10.1007/s00394-013-0516-8	197	15.15
Qu JL, 2020, Int J Biol Macromol	10.1016/j.ijbiomac.2020.05.196	139	19.86	Oz HS, 2013, Front Immunol	10.3389/fimmu.2013.00132	193	13.79
Ore A, 2019, Medicina-Lithuania	10.3390/medicina55020026	139	17.38	Klatsky AL, 2006, Arch Intern Med	10.1001/archinte.166.11.1190	192	9.14

**FIGURE 6:**
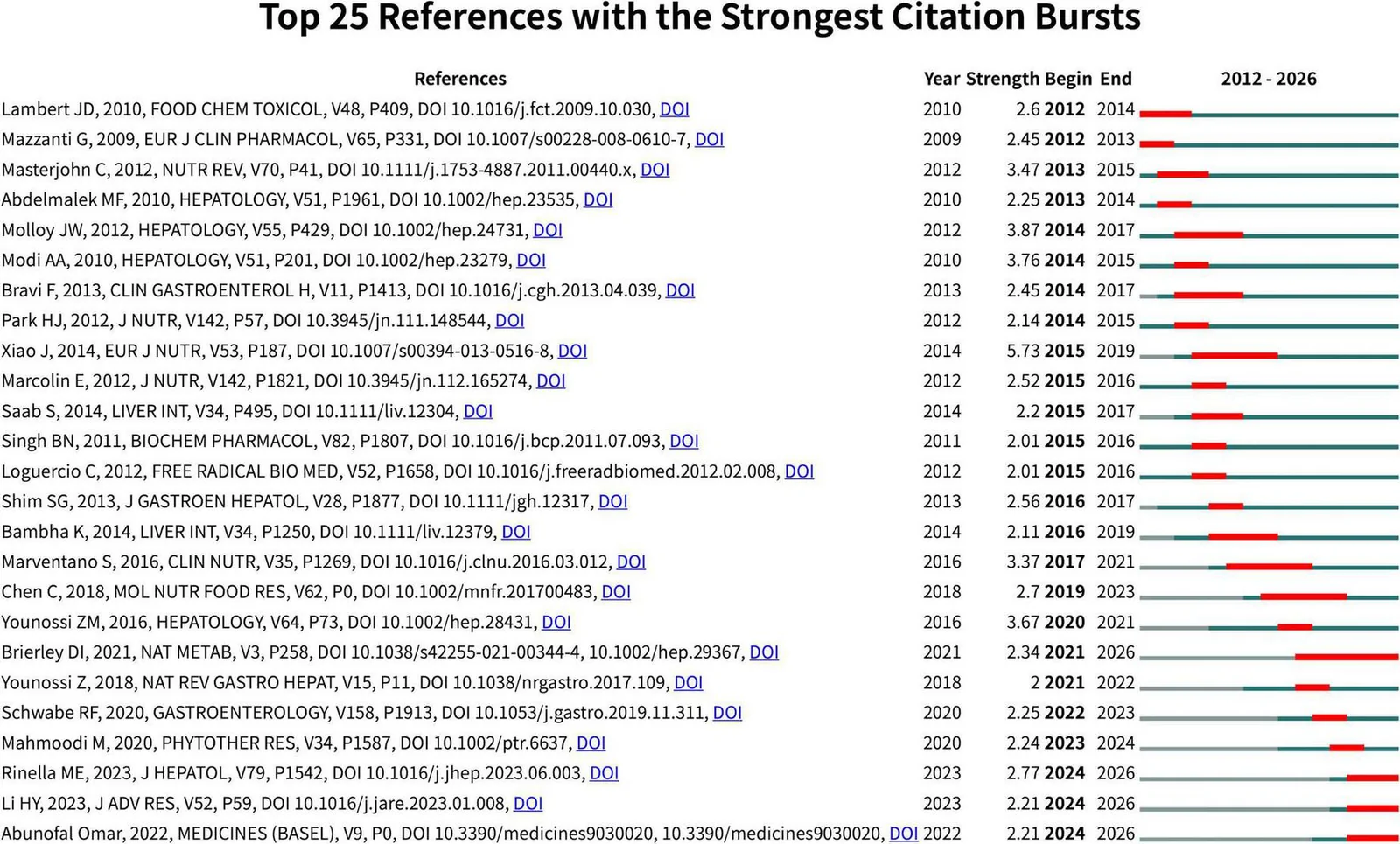
Top 25 references with the strongest citation bursts in tea for liver fibrosis.

To identify prominent citation burst patterns in the field of tea for liver fibrosis treatment within the WoSCC database, we employed CiteSpace to detect 25 references with notable citation bursts based on specific criteria, of which 25 are displayed in Figure 6. The titles of these citations, along with their respective DOIs, are listed in Annex 3. The citation burst intensities of these 25 documents range from 2 to 5.73.

### 3.4 Keyword clusters and evolution of themes

Keyword clustering provides a rapid approach to identifying major themes and research directions within a specific field. In this study, VOSviewer was employed to extract 1,595 keywords from the WoSCC database and 7,519 keywords from the Scopus database. Table 5 presents the top 20 keywords.

**TABLE 5:** The top 20 keywords.

WoSCC database			Scopus database		
Rank	Keywords	Count	Rank	Keywords	Count
1	Oxidative stress	88	1	Human	254
2	Nafld	66	2	Article	235
3	Inflammation	48	3	Nonhuman	204
4	Nash	47	4	Male	179
5	Epigallocatechin gallate	39	5	Controlled study	161
6	Expression	35	6	Animal	130
7	Hepatocellular carcinoma	35	7	Unclassified drug	116
8	Insulin resistance	35	8	Animal experiment	111
9	Hepatic stellate cell	30	9	Animal model	107
10	Antioxidant	27	10	Drug effect	96
11	nf-kappa-b	27	11	Animal tissue	94
12	Coffee	26	12	Metabolism	94
13	Mechanism	25	13	Review	94
14	Risk	25	14	Alanine aminotransferase	93
15	Activation	24	15	Oxidative stress	93
16	Disease	24	16	Adult	85
17	Cirrhosis	23	17	Female	85
18	Polyphenol	23	18	Antioxidant	83
19	Liver disease	22	19	Aspartate aminotransferase	82
20	Green tea polyphenol	20	20	Priority journal	81

**FIGURE 7:**
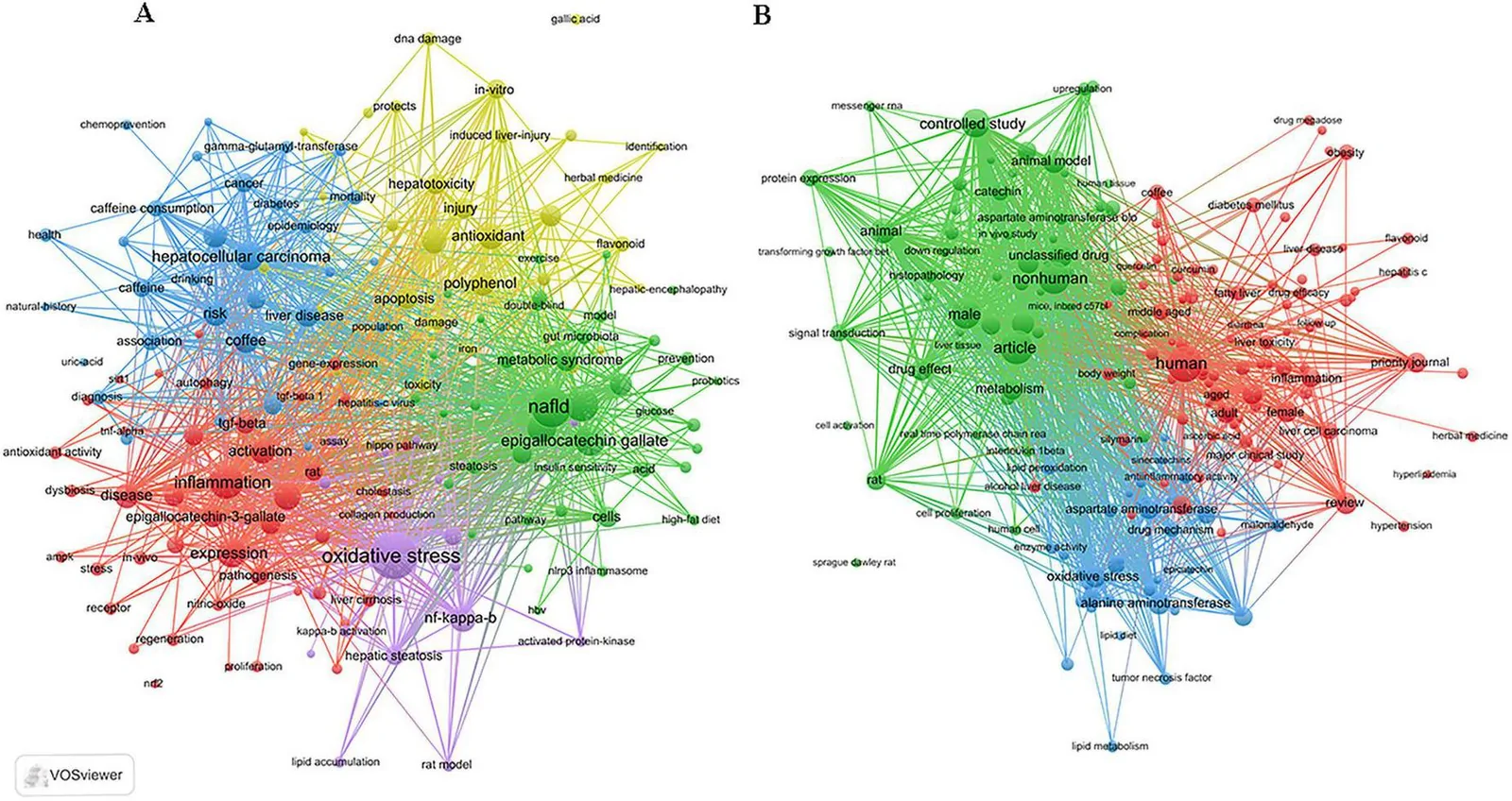
Keyword co-occurrence map of publications on tea for liver fibrosis (**A** from the WoS database; **B** from the Scopus database).

Based on these data, we selected 209 keywords appearing ≥ 3 times in WoSCC and 209 keywords appearing ≥ 15 times in Scopus to generate keyword cluster maps (Figures 7A,B). In WoSCC, five major clusters were identified and analyzed, each containing ≥ 17 keywords: EGCG/tea polyphenols in core pathological mechanisms of liver fibrosis (Red cluster): 44 keywords, including inflammation, expression, and hepatic stellate cell. EGCG/tea polyphenols in preclinical intervention for Non-Alcoholic Fatty Liver Disease (NAFLD)/Non-Alcoholic Steatohepatitis (NASH)-related liver fibrosis (Green cluster): 40 keywords, including NAFLD, NASH, and epigallocatechin gallate. Tea/coffee consumption and epidemiological associations with liver fibrosis, cirrhosis, and HCC risk (Blue cluster): 32 keywords, including hepatocellular carcinoma, coffee, and risk. Hepatoprotective and anti-fibrotic pharmacological screening of natural bioactive components in tea (Yellow cluster): 30 keywords, including antioxidant, mechanism, and polyphenol. Catechin targeting of oxidative stress-inflammation axis in liver fibrosis amelioration (Purple cluster): 17 keywords, including oxidative stress, NF-kappa-B, and hepatic steatosis. In Scopus, three major clusters were identified and analyzed, each containing ≥ 37 keywords: Clinical and epidemiological studies of tea and bioactive constituents in chronic liver diseases related to liver fibrosis (Red cluster): 88 keywords, including human, review, and adult. Core pharmacological and molecular mechanisms of EGCG/catechins against liver fibrosis (Green cluster): 60 keywords, including article, nonhuman, and male. Pharmacodynamic evaluation and antioxidant/anti-inflammatory mechanisms of tea-derived natural products in hepatoprotection and anti-fibrosis (Blue cluster): 37 keywords, including alanine aminotransferase, oxidative stress, and aspartate aminotransferase (complete list in Annex 4).

**FIGURE 8:**
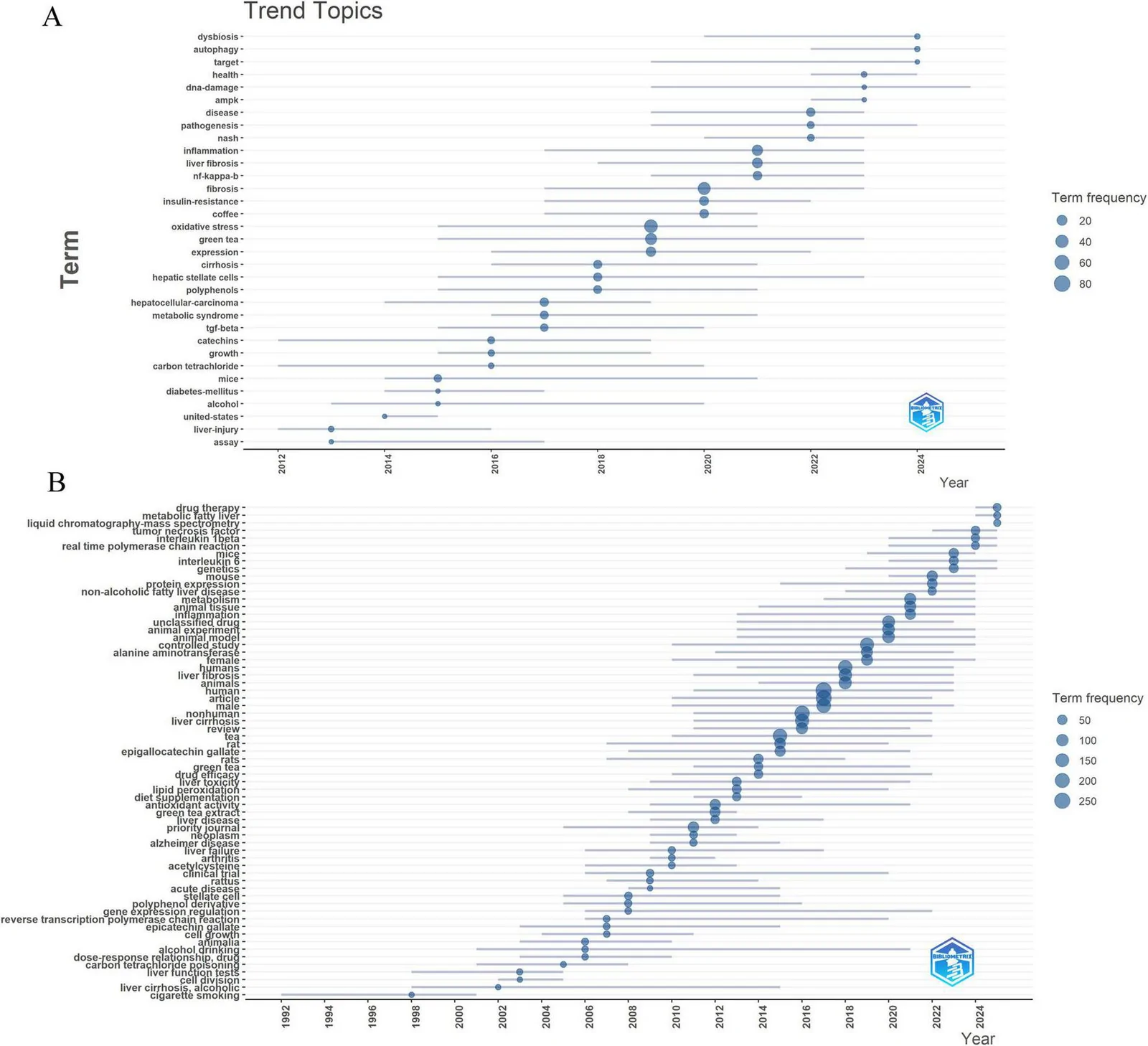
Trend topics on tea for liver fibrosis (**A** from the WoS database; **B** from the Scopus database).

To analyze developmental trends across both databases, dynamic thematic evolution maps were constructed using the bibliometrix package in the R programming environment (Figures 8A,B). The research trajectory sequentially evolved through three phases: (1) Preliminary pharmacological exploration (1998–2010), focusing on foundational validation of hepatoprotective and anti-fibrotic activities of tea and tea extracts; (2) Molecular mechanism deepening (2011–2019), centered on catechins and tea polyphenols as core bioactives, with in-depth investigation into oxidative stress, TGF-β signaling, and hepatic stellate cell activation pathways; and (3) Clinical scenario expansion (2020–2026), concentrating on NASH-related liver fibrosis, intestinal dysbiosis, novel AMPK/autophagy mechanisms, and combined intervention strategies. Looking ahead, the future of tea in liver fibrosis treatment will be closely tied to: the construction of high-quality clinical evidence through multicenter randomized controlled trials (addressing the current evidence gap dominated by animal studies); refinement of gut-liver axis-mediated microbiota regulatory mechanisms; development of novel targets such as AMPK/autophagy; optimization of bioavailability for tea bioactive constituents; and design of synergistic multi-natural product intervention protocols. Collectively driving the field from basic mechanistic research toward precision and personalized clinical applications.

### 3.5 Comprehensive analysis of hotspots

In summary, through the aforementioned comprehensive analyses, we identified four major emerging research hotspots in tea for liver fibrosis treatment: (1) core molecular mechanisms and multi-target synergistic effects of tea bioactive constituents against liver fibrosis; (2) gut-liver axis modulation through intestinal microbiota regulation by tea bioactives; (3) translational research on combined anti-fibrotic effects of tea with other natural products/dietary factors; and (4) bioavailability optimization strategies for tea bioactive constituents.

## 4 Discussion

### 4.1 General information

We retrieved 275 and 391 relevant articles from the WoSCC and Scopus databases, respectively. Overall, publication output in this field demonstrated an upward trend. By publication volume, Nutrients and Frontiers in Pharmacology ranked as the top two journals. In terms of citation frequency, Hepatology ranked first by a substantial margin, reaffirming its position as the primary conduit for disseminating influential research in this field.

### 4.2 Hotspots and development trends

#### 4.2.1 Core molecular mechanisms and multi-target synergistic effects of tea bioactive constituents against liver fibrosis

Tea polyphenols constitute the core functional components in the prevention and treatment of liver fibrosis. These bioactives are typified by EGCG from green tea. They further encompass monomeric compounds, including theaflavins, theabrownins, and flavonoids, as well as complex bioactive systems such as composite tea extracts and tea-derived extracellular vesicles (TEVs). Owing to divergent fermentation processes, the compositional profiles of active constituents differ markedly across tea categories. Green and white teas are abundant in free catechins such as EGCG (12); black and dark teas undergo extensive fermentation that converts the majority of catechins into theaflavins and theabrownins (13). Dihydromyricetin (DHM), E Se tea extract (ESE), and Penthorum Chinense Pursh Leaf Tea (PLT) are characterized by dihydromyricetin and various flavonoid glycosides as their signature active principles (14, 15). Furthermore, TEVs contain highly abundant internal microRNAs (miRNAs). By virtue of this cargo, they serve as a unique bioactive delivery platform and expand the functional modalities of tea-derived agents (16, 17). Collectively, these monomers and extracts target HSC activation. They exert protective effects through antioxidant, anti-inflammatory, and multi-pathway regulatory mechanisms. Nevertheless, their specific molecular targets and signaling pathways exhibit distinct monomer-specific signatures.

In recent years, emerging studies have systematically elucidated the core pathological mechanisms through which tea bioactive constituents exert anti-hepatic fibrotic effects. These mechanisms are summarized as follows.

(1) Targeted inhibition of HSC activation, proliferation, and migration. The transdifferentiation of quiescent HSCs into myofibroblasts represents the initiating event of liver fibrosis. Diverse tea-derived bioactive compounds significantly downregulate the HSC activation marker α-smooth muscle actin (α-SMA). They reduce the deposition of extracellular matrix (ECM) components, including collagen types I and III. They also suppress HSC proliferation, migration, and contractility (18–20). EGCG blocks HSC transdifferentiation through multiple distinct signaling cascades. On one hand, it suppresses both the TGF-β-induced transcription and enzymatic activity of sphingosine kinase 1 (SphK1). This interrupts the pro-fibrotic SphK1/S1P/MAPK signaling axis (7). On the other hand, RNA sequencing analyses have demonstrated that EGCG downregulates phospholipase C epsilon 1 (PLCE1). This restraint reduces IP_3_-mediated Ca^2+^ release from the endoplasmic reticulum. Consequently, cytoskeletal remodeling is impaired, and the migratory capacity of HSCs is reduced (21). Concurrently, EGCG upregulates the circular RNA mmu_circRNA_011775. This leads to transcriptional silencing of collagen and other pro-fibrotic genes (22). High-purity theaflavin monomers activate the PKA-LKB1-AMPK-GSK3β pathway. This activation stabilizes nuclear factor erythroid 2-related factor 2 (Nrf2) and eliminates reactive oxygen species (ROS). Oxidative stress-triggered HSC activation is thereby abrogated (23). TEVs can be internalized by hepatocytes and HSCs. Their enriched cargo of miR-44 dose-dependently inhibits Smad2/3. This inhibition reverses the fibrotic phenotype of HSCs (17). Additionally, several other tea-derived agents exert inhibitory effects on α-SMA expression and collagen deposition. These include DHM from *Ampelopsis grossedentata* (vine tea), theabrownins, dark tea extracts, ESE flavonoids, isochlorogenic acid A (ICAA), white tea polyphenols, and compound herbal tea preparations. By suppressing these markers, they restrict excessive HSC activation.

(2) Modulation of the canonical TGF-β/Smad pro-fibrotic signaling pathway. The TGF-β/Smad cascade serves as the central signaling axis driving HSC activation. Nearly all tea bioactive ingredients target this pathway. EGCG, theaflavin extracts from dark tea, theabrownin (HT) from black tea, ESE, ICAA, and TEVs all downregulate TGF-β1 expression. They also inhibit Smad2/3 phosphorylation. Consequently, Smad nuclear translocation is prevented, and the transcription of fibrotic genes is blocked. Notably, EGCG achieves dual suppression of canonical and non-canonical fibrotic pathways. It simultaneously blocks the transcriptional activation of SphK1. This activation is mediated by both upstream Smad3 and downstream MAPK signaling (7). TEVs carry miR-44 to directly target Smad2/3 mRNA, inhibiting pathway activation at the post-transcriptional level (17). Combined administration of theaflavins and EGCG with silymarin synergistically reduces phosphorylated Smad1/2 (p-Smad1/2). This combination further attenuates ECM biosynthesis (5, 24). DHM from *Ampelopsis grossedentata* concurrently represses TGF-β1. It also activates anti-apoptotic pathways in hepatocytes. These actions indirectly attenuate HSC proliferation driven by liver injury signals (14). Furthermore, Xiasangju (XSJ) can upregulate the endogenous inhibitory protein Smad7 to antagonize TGF-β/Smad signal transduction (25).

(3) Regulation of antioxidant stress pathways. Oxidative stress functions as an upstream initiator and amplifier of HSC activation. Tea polyphenols and flavonoids exert dual functions. They directly scavenge free radicals and activate endogenous antioxidant pathways. EGCG, white tea, dark tea, ESE, and theaflavin monomers elevate the levels of antioxidant molecules. These include superoxide dismutase (SOD), catalase (CAT), reduced glutathione (GSH), heme oxygenase-1 (HO-1), and NAD§H:quinone oxidoreductase 1 (NQO1). Concurrently, they reduce lipid peroxidation products, including malondialdehyde (MDA) and 4-hydroxynonenal (4-HNE). Theaflavins inhibit glycogen synthase kinase-3β (GSK3β) activity through the PKA-LKB1 axis. This relieves GSK3β-mediated Nrf2 degradation. Nrf2 nuclear translocation is thereby facilitated, systematically activating antioxidant defense responses (23). ESE and ICAA directly upregulate Nrf2 protein expression (15, 26). Green tea polyphenols (GTP) and fermented dark tea modulate glutathione and ferroptosis-related metabolic pathways. They thereby ameliorate oxidative metabolic disorders induced by xenobiotics and high-fat diets. In turn, this mitigates persistent oxidative hepatic damage (24, 27).

(4) Inhibition of NF-κB-mediated inflammatory pathways. Chronic inflammation sustains the release of tumor necrosis factor-α (TNF-α), interleukin-1β (IL-1β), and IL-6. This constructs a pro-fibrotic hepatic microenvironment. Black tea extracts (HT), DHM, ESE, ICAA, and EGCG-containing compound preparations all block NF-κB cascade activation. They suppress IKKα/β phosphorylation and retard IκBα degradation. These actions hinder p65 nuclear translocation. Consequently, the production of multiple pro-inflammatory cytokines is drastically decreased. In HBV-induced fibrotic models, EGCG promotes autophagy-dependent degradation of the damage-associated molecular pattern high-mobility group box 1 (HMGB1) in hepatocytes. It also restrains the activation of Kupffer cell NLRP3 inflammasomes. These effects indirectly lower IL-1β secretion (28). Caffeine and EGCG modulate distinct inflammatory signaling axes. Caffeine preferentially regulates JAK-STAT and insulin-mediated inflammatory responses. EGCG primarily targets lipid-associated inflammation (29).

(5) Modulation of non-canonical pathways including MAPK, AMPK, calcium signaling, and lipid metabolism. Beyond the core TGF-β/Smad, NF-κB, and Nrf2 pathways, tea bioactive ingredients regulate multiple auxiliary signaling axes. They thereby form a multi-target regulatory network. (i) MAPK pathway: Theaflavins (24), theabrownins (8), ICAA (26) suppress the phosphorylation of extracellular signal-regulated kinase (ERK), c-Jun N-terminal kinase (JNK), and p38. This blocks downstream fibrotic gene transcription. (ii) AMPK pathway: Theaflavins (24) and ICAA (26) activate AMPK. This concurrently inhibits GSK3β and MAPK. Combined antioxidant and anti-inflammatory effects are thereby elicited. (iii) Calcium signaling: EGCG represses the PLCE1-IP_3_ cascade to decrease intracellular Ca^2+^ concentration and block HSC migration (21). (iv) Lipid metabolism: Fermented dark tea upregulates cholesterol 7α-hydroxylase (CYP7A1) to accelerate cholesterol catabolism (30). Meanwhile, GTP (27) and PLT (31) ameliorate triglyceride and cholesterol accumulation. This mitigates NAFLD-associated liver fibrosis. (v) Autophagy and non-coding RNAs: EGCG modulates hepatocyte autophagy. It also regulates the expression of circular RNAs (circRNAs). Both processes actively participate in the regulation of fibrogenesis (22). (vi) Theaflavin-rich extracts suppress aberrant overexpression of matrix metalloproteinase-2 and -9 (MMP-2/MMP-9). This restores the dynamic equilibrium between ECM synthesis and degradation (24).

The TGF-β/Smad axis constitutes the core therapeutic target and rate-limiting step of EGCG-mediated anti-fibrotic effects, directly driving HSC activation, the final common pathway of liver fibrosis. Nrf2-mediated antioxidant and NF-κB-mediated anti-inflammatory pathways serve as upstream auxiliary mechanisms that indirectly suppress TGF-β signaling activation by attenuating oxidative stress and inflammation. Meanwhile, the AMPK/autophagy pathway operates as a metabolic regulatory auxiliary mechanism in contexts of metabolic stress such as NAFLD. This hierarchical framework is concordant with machine learning-based meta-analytic (32) findings demonstrating that EGCG modulates the ROS phenotype through NF-κB/Nrf2, while specifically ameliorating liver fibrosis via TGF-β/Smad.

#### 4.2.2 Gut-liver axis modulation: intestinal microbiota regulation by tea bioactives in liver fibrosis amelioration

The intestine and liver constitute a bidirectional communication network, the gut–liver axis. They are linked via the portal circulation and enterohepatic bile acid cycling (33). Under conditions of chronic hepatic injury, intestinal dysbiosis frequently ensues. It is accompanied by disruption of epithelial tight junctions and a consequent increase in intestinal permeability (34). Gram-negative bacteria then release large quantities of endotoxins, notably lipopolysaccharide (LPS), into the bloodstream (35). These translocate via the portal vein to the liver. There, they chronically activate Kupffer cells to release pro-inflammatory mediators, including tumor necrosis factor-α (TNF-α) and interleukin-1β (IL-1β) (33, 36). These cytokines stimulate HSC activation and accelerate collagen deposition. They thereby drive the progressive worsening of liver fibrosis.

Moreover, microbial metabolites function as inter-organ signaling molecules. These include bile acids, intestinal 5-hydroxytryptamine (5-HT), and short-chain fatty acids (SCFAs) (37, 38). They relay pathological signals from the gut to the liver. This establishes a vicious cycle: intestinal injury leads to hepatic inflammation, which in turn aggravates fibrosis. Therefore, restoring intestinal homeostasis and interrupting deleterious signaling through the gut–liver axis represents a critical strategic target for preventing and treating liver fibrosis.

Multiple tea extracts and selenium-enriched specialty teas can simultaneously reshape intestinal microbiota architecture and restore mucosal barrier integrity. They thereby sever endotoxin-mediated hepatic injury pathways. Fermented dark tea extract (DTE) significantly upregulates the expression of colonic tight junction proteins. These include Occludin, zonula occludens-1 (ZO-1), and Claudin-1 (6). It also alleviates mucosal inflammatory infiltration and reduces intestinal permeability. Consequently, LPS entry into the circulation is decreased. Ampelopsis grossedentata tea flavonoids (AG) can remodel bile duct ligation (BDL)-induced dysbiosis. This has been confirmed by 16S rDNA sequencing (39). Specifically, AG decreases the Firmicutes-to-Bacteroidetes ratio. It also enriches beneficial bacteria such as Dubosiella and Parasutterella. At the same time, it suppresses the proliferation of pro-fibrotic taxa, including Ruminococcaceae (39). Selenium-enriched green tea (SeT) leverages the synergism between tea polyphenols and selenium. It reduces the expression of tryptophan hydroxylase 1 (TPH1) (38). This diminishes intestinal 5-HT secretion. Consequently, 5-HT-mediated hepatic injury signals are blocked at their intestinal origin. Collectively, these tea bioactives enhance the abundance of beneficial genera such as Bacteroides. They inhibit pathogen overgrowth. They also reinforce the intestinal epithelial barrier to attenuate endotoxin delivery to the liver.

Tea active ingredients utilize intestinal microbiota, bile acids, and gut-derived signaling molecules as intermediary mediators. They form an integrated regulatory chain: tea constituents modulate intestinal homeostasis, which in turn ameliorates hepatic pathology. This axis can be categorized into three core regulatory pathways.

First, the farnesoid X receptor (FXR)/Takeda G protein-coupled receptor 5 (TGR5)–bile acid metabolic pathway. Fermented DTE concurrently upregulates FXR and TGR5 expression in both the colon and liver (6). It also downregulates ATP-binding cassette subfamily F member 1 (ABCF1). These actions correct bile acid metabolic disorders. The restoration of bile acid homeostasis suppresses the release of hepatic pro-inflammatory factors. It also ameliorates sinusoidal capillarization. Additionally, it inhibits vascular endothelial growth factor A (VEGFA)-mediated pathological angiogenesis. Consequently, pathological crosstalk between liver sinusoidal endothelial cells (LSECs) and HSCs is attenuated. Fibrogenesis is thereby interrupted at multiple levels.

AG can likewise correct bile acid metabolic disturbances in BDL models (39). It elevates protective bile acid species such as chenodeoxycholic acid (CDCA) and taurochenodeoxycholic acid (TCDCA). These changes mitigate hepatic inflammation and collagen deposition via bile acid-mediated gut–liver signaling.

Second, Intestinal 5-HT–hepatic 5-HTR signaling axis. SeT inhibits duodenal TPH1 (38). This reduces intestinal 5-HT generation. Simultaneously, it downregulates hepatic 5-HT2A/2B receptors and monoamine oxidase A (MAO-A) expression. These actions block portal 5-HT-induced HSC proliferation. They also prevent the maintenance of pro-fibrotic phenotypes. Notably, selenium and tea polyphenols exert synergistic antioxidant and anti-fibrotic effects. These amplify the protective actions.

Third, the microbiota-mediated oxidative–inflammatory suppression cascade. Multi-omics data for AG demonstrate that microbiota remodeling alters metabolic profiles (39). These profiles include SCFAs, lipids, and other metabolites. The remodeling results in reduced harmful metabolites and enriched protective metabolites. These changes alleviate systemic oxidative stress. They also improve the hepatic inflammatory microenvironment. Consequently, HSC activation is indirectly suppressed.

Although standard validation techniques such as fecal microbiota transplantation (FMT) have not yet been comprehensively conducted in gut–liver axis-related tea research, microbiota–metabolite correlation analyses have provided substantial evidence. Intestinal microbiota constitute an essential intermediate medium through which tea bioactivity exerts hepatoprotective effects.

In summary, diverse tea active ingredients operate through the gut–liver axis. They exert multi-link synergistic intervention against liver fibrosis. Upstream, they restore the intestinal barrier and reshape gut microbiota. Midstream, they regulate gut-derived signaling molecules such as bile acids and 5-HT. Downstream, they block hepatic oxidative stress, inflammation, and HSC activation. This integrated, axis-dependent modulation underlies the therapeutic efficacy of tea constituents in suppressing liver fibrosis.

Notably, the regulatory effects of tea polyphenols on the intestinal microbiota share certain functional similarities with those of traditional dietary fibers and prebiotics. Both can reshape microbial composition, enrich beneficial genera, and promote SCFA production (40). However, tea polyphenols also exhibit distinctive specificities that differentiate them from typical prebiotic interventions. First, following microbial biotransformation, tea polyphenols yield bioactive phenolic acids that exert direct anti-inflammatory and antioxidant effects upon entering the liver. Second, tea polyphenols concurrently modulate intestinal microbial architecture, barrier integrity, bile acid homeostasis, and 5 HT signaling pathways, thereby conferring substantial mechanistic diversity.

Beyond this, direct HSC inhibition is achieved via suppression of the MDM2/MUC5AC-mediated TGF β1/Smad (41) and PLCE1/IP3/Ca^2+^ (21) cascades, offering well defined molecular targets and straightforward pharmacodynamic assessment. Nevertheless, this direct route is severely constrained by the extremely low oral bioavailability of EGCG. By contrast, the indirect gut liver axis mechanism exploits the high local concentrations of unabsorbed tea polyphenols within the intestinal lumen, enabling direct interactions with the gut microbiota and epithelial barrier. Therefore, from a translational feasibility standpoint, the indirect mechanism may provide a more practical avenue that fully capitalizes on the pharmacokinetic properties of tea polyphenols. However, the durability of indirect effects may be compromised by interindividual variability in baseline microbial composition. Meanwhile, direct HSC inhibition affords more predictable target engagement, although it necessitates frequent dosing due to the short half-life of EGCG.

Collectively, direct HSC inhibition and indirect gut liver axis modulation constitute a dual safeguard underlying the anti-fibrotic efficacy of tea polyphenols. The former supplies mechanistically explicit targets, whereas the latter offers a pharmacokinetically viable strategy that bypasses the constraint of low systemic bioavailability. The synergistic integration of both approaches, rather than exclusive selection of either, represents the most reliable and sustainable therapeutic strategy.

#### 4.2.3 Combined anti-fibrotic interventions: tea with other natural products and dietary factors

Numerous large cross-sectional and long-term prospective cohorts across multiple regions consistently report significant inverse correlations between regular tea/caffeine intake and the risk of liver fibrosis, cirrhosis and hepatocellular carcinoma. Some cohorts further identify synergistic protective effects of combined tea and coffee consumption. Although confounders such as alcohol use, viral hepatitis, age, gender and genetics partly dilute these protective signals, tea and coffee remain independent protective predictors after full multivariate adjustment.

However, two major methodological flaws limit the reliability of such observational associations. First, even comprehensive covariate adjustment cannot rule out residual bias from unmeasured lifestyle and metabolic factors, weakening causal inference from these population data. Second, existing Mendelian randomization (MR) (42, 43) analyses only deliver conflicting causal results for NAFLD onset, and no large-scale robust MR evidence specifically uses liver stiffness, histology or FIB-4 fibrosis scores as primary endpoints to verify tea’s causal anti-fibrotic effect. Future MR research centered on liver fibrosis grading is needed to resolve this causal uncertainty.

We systematically retrieved clinical research articles on tea beverages and dietary supplements for liver fibrosis. Specifically, we included 16 articles from the WoSCC and PubMed databases. Their clinical trial designs, efficacy endpoints, and follow-up outcomes are summarized in Table 6. More detailed content regarding clinical studies is presented in Annex 5.

**TABLE 6:** Summary of clinical studies on tea and its active ingredients for liver fibrosis intervention.

Study type	Tested ingredient	Study population	Sample size	Key efficacy results	Main conclusions	References
Multicenter Phase I dose-escalation trial	Polyphenon E (core component: EGCG)	Patients with Child-Pugh A cirrhosis, NASH/Hepatitis C Virus (HCV)-related, at high risk for HCC	13 completed treatment	1. Intrahepatic γ-OHPdG decreased in all dose groups except one subject; 2. Improved liver stiffness and hepatic steatosis in some patients.	EGCG was safe and reduced hepatic oxidative damage in cirrhotic patients; 1200 mg/day is recommended for Phase II trials.	(56)
Cross-sectional population epidemiological survey	Tea (daily tea consumption)	Adults aged ≥ 18 years in Guangdong, China, excluding viral hepatitis, etc.	7,287 participants	Daily tea consumption was an independent protective factor against MASLD in Han population	Daily tea consumption reduces hepatic steatosis risk. MASLD burden.	(69)
Cross-sectional clinical study	Caffeinated tea, coffee, and soda	Chronic HCV patients at the U.S. Veterans Affairs Medical Center, untreated with antiviral therapy.	342 with advanced fibrosis, 568 with mild/no fibrosis	Caffeine intake ≥ 100 mg/day and ≥ 1 cup/day of caffeinated tea reduced advanced fibrosis risk	Low-dose caffeine reduces advanced fibrosis risk in men with chronic HCV.	(70)
Prospective population-based cohort study	Coffee, tea (green, black, herbal)	General Dutch population aged ≥ 45 years without severe liver disease	2,424 participants	Frequent coffee reduced fibrosis risk; herbal tea lowered liver stiffness; green/black tea showed no association.	Frequent coffee and herbal tea intake reduce subclinical fibrosis risk; longitudinal validation needed.	(71)
Genome-wide association study (GWAS)	Tea, coffee	General Taiwanese population aged 30–70 years without severe liver disease	59,448 included; 1,476 with high fibrosis risk	Daily tea reduced fibrosis risk; coffee had no effect. Age, male sex, alcohol, and viral hepatitis were independent risk factors.	Daily tea protects against liver fibrosis in Taiwanese; genetics drive initiation, diet modulates progression.	(72)
Randomized double-blind placebo-controlled Phase I trial	catechin green tea (EGCG)	Patients with imaging-confirmed NAFLD	7 in high-catechin group, 5 in low-catechin group, 5 in placebo group	High-catechin group improved body fat, steatosis, ALT, and oxidative stress	High-catechin green tea: a potential dietary intervention for NAFLD-related fibrosis.	(73)
Large-scale prospective cohort study	Tea (daily dietary intake)	UK adults (40–70 years) without chronic liver disease or cancer.	372,492 enrolled; 3,527 NAFLD, 1,643 cirrhosis, 669 HCC cases.	Higher tea intake was inversely associated with NAFLD, cirrhosis, and HCC risk; high-intake healthy diet group had 15% lower cirrhosis risk.	Daily tea consumption protects against chronic liver disease, reducing fibrosis and end-stage liver disease risk.	(9)
Prospective cross-sectional epidemiological survey	Tea (daily dietary intake)	Guangdong residents aged ≥ 18 years, excluding viral hepatitis, etc.	7,388 included; 2,240 with MASLD	Increased tea intake inhibits fibrosis progression. Insulin resistance independently predicts fibrosis in MASLD.	Daily tea intake reduces steatosis risk and may inhibit fibrosis progression in Han Chinese.	(74)
Cross-sectional clinical study	Green tea	Cirrhotic patients aged ≥ 18 years, excluding liver transplant, etc.	Child-Pugh A: 45; Child-Pugh B: 17	Green tea was the main dTAC contributor; dTAC did not correlate with cirrhosis severity or liver function.	Green tea-based high-antioxidant diet may improve muscle mass and strength in cirrhotic patients.	(75)
Longitudinal observational cohort study	Tea, coffee	HIV-HCV co-infected patients aged ≥ 18 years, excluding liver transplant, etc.	1,019 enrolled; 147 advanced fibrosis cases.	High coffee intake reduced advanced fibrosis risk; combined tea and coffee showed synergistic anti-fibrotic effects.	Daily tea and coffee reduce advanced fibrosis risk in HIV-HCV patients; combined intake exerts synergistic anti-fibrotic effects.	(76)
Randomized double-blind placebo-controlled Phase II trial.	Standardized green tea polyphenol extract	NASH-related liver fibrosis (F1–F3)	60 in green tea polyphenol group, 60 in placebo group	Green tea polyphenols improved liver stiffness, fibrosis scores, fibrosis reversal rate exceeded placebo.	800 mg/day green tea polyphenols improves NASH-related fibrosis via anti-inflammatory and antioxidant effects	(77)
Multicenter open-label dose-escalation Phase I trial	High-purity EGCG	Child-Pugh A cirrhosis	36 screened, 18 enrolled, 17 completed	Plasma EGCG rose dose-dependently; 400 and 600 mg groups improved liver stiffness, FIB-4, and oxidative stress	High-purity EGCG is safe with favorable pharmacokinetics in Child-Pugh A cirrhosis; 400 mg/day recommended for Phase II trials.	(78)
Large-scale prospective cohort study	Tea (green, black, oolong, etc.)	Chinese adults (30–79 years) without chronic liver disease or cancer.	2,567 cirrhosis and 1,028 HCC cases recorded	Daily tea reduced cirrhosis and HCC risk with a dose-response relationship; green tea showed the strongest protection	Daily tea independently protects against liver fibrosis, significantly reducing significant fibrosis risk.	(10)
Cross-sectional population study	Tea (green, black, herbal)	US adults ( ≥ 20 years) without liver disease, severe illness, or cancer.	2,135 with NAFLD, 1,248 with significant fibrosis	Daily tea reduced fibrosis risk and improved liver function in NAFLD	Daily tea independently protects against liver fibrosis in US adults, significantly reducing significant fibrosis risk.	(79)
Prospective cohort study	Tea	Japanese adults (40–79 years) without chronic liver disease or cancer.	1,872 cirrhosis and 721 HCC cases recorded	Daily tea reduced cirrhosis and HCC risk, inhibited fibrosis progression.	Daily tea independently protects against cirrhosis and HCC, reducing fibrosis and end-stage liver disease risk.	(80)
Retrospective cohort study	Tea (green, black, oolong)	Taiwanese adults ( ≥ 20 years) without chronic liver disease or cancer.	1,245 cirrhosis and 568 HCC cases recorded	Daily tea reduced cirrhosis and HCC risk; green tea showed the strongest protection, stable in high-risk groups.	Daily tea independently protects against cirrhosis and HCC in Taiwanese adults, reducing fibrosis and end-stage liver disease risk.	(81)

Collectively, existing clinical data highlight three prominent advantages of tea-derived polyphenols for the dietary management of liver fibrosis. First, they exhibit an excellent safety profile. Oral administration is associated only with mild gastrointestinal side effects. No severe hepatorenal toxicity has been reported. Thus, these polyphenols are suitable for long-term maintenance intervention in chronic liver diseases. Second, they possess broad applicability. They demonstrate efficacy across NAFLD, viral hepatitis-related fibrosis, and early-stage cirrhosis. Third, they confer pleiotropic benefits. They simultaneously ameliorate hepatic steatosis, insulin resistance, and systemic oxidative inflammation. They also mitigate cirrhosis-related complications. In addition, they contribute to the primary prevention of HCC.

Nevertheless, several bottlenecks currently hinder the clinical translation of tea-based dietary interventions. (i) There is substantial heterogeneity in population epidemiology, including geographic, ethnic disparities, inconsistent tea categories, brewing modes and daily intake standards. Notably, the 16 included studies mainly cover unfermented green tea, semi-fermented oolong tea and fully fermented black tea, while clinical data on post-fermented dark tea are scarce. Preclinical studies show varied polyphenol compositions and hepatoprotective pathways across the three tea types, yet uniform head-to-head comparative experiments are lacking, which cannot confirm their distinct clinical efficacy. Existing epidemiological evidence is inconsistent: Western multi-ethnic cohorts link high black tea intake to lower FIB-4 scores, while Asian studies only associate green tea with reduced fatty liver risk; oolong tea samples are too limited for reliable subgroup analysis. In addition, dietary questionnaires carry subjective bias, weakening cross-study reproducibility. Overall, divergent outcome indicators and uneven sample distribution break the evidence chain of tea fermentation gradients, and current population data cannot accurately quantify the separate anti-fibrotic effects of different teas. (ii) High-quality clinical evidence remains scarce. Existing intervention trials are predominantly small in sample size. They also adopt single-center designs and short durations (12–24 weeks). Large-scale, multi-center Phase III randomized controlled trials (RCTs) with long-term follow-up exceeding three years are lacking. Moreover, validation against hard endpoints, such as cirrhosis decompensation and HCC incidence, remains insufficient. (iii) Personalized intervention frameworks are absent. Differentiated dosing regimens for tea or polyphenol supplementation have not been established based on etiology, fibrosis stage, or individual metabolic phenotypes. This gap precludes precision dietary interventions. (iv) Research on combination therapies is insufficient. Few clinical trials examine tea polyphenols combined with coffee, silymarin, selenium or curcumin. Synergistic anti-fibrotic effects are only observed in animal models without clinical validation. Existing studies also lack analyses of optimal dose ratios, CYP450 metabolic interactions and cumulative hepatotoxicity risks. Specialized pharmacology and toxicology trials are required to quantify safe and effective combined dosing schemes.

However, given the substantial heterogeneity across existing trials in target populations, disease stages, outcome indicators, and categories of tea with varying fermentation degrees, the optimal intervention timing, applicable fibrosis stage, unified primary endpoint, as well as the differential hepatoprotective effects of different fermented teas should be rigorously defined through future systematic reviews, meta-analyses, or expert consensus, rather than directly inferred merely from this bibliometric overview.

#### 4.2.4 Bioavailability optimization strategies for tea bioactive constituents

EGCG is the most abundant and pharmacologically active catechin in green tea. It possesses potent antioxidant, anti-inflammatory, and anti-fibrotic properties (44–46). However, its clinical translation is substantially hindered by unfavorable physicochemical characteristics and pharmacokinetic limitations.

First, physicochemical and stability constraints. EGCG contains multiple phenolic hydroxyl and aromatic groups. These structural features confer high polarity and poor lipophilicity (47). Consequently, it exhibits low intestinal membrane permeability. Its transcellular absorption is also inefficient, despite modest aqueous solubility (48). More importantly, EGCG is chemically labile under high temperature and neutral or alkaline conditions (pH > 6). It is also sensitive to light and oxygen. These factors lead to rapid oxidative degradation prior to absorption (49, 50).

Second, pharmacokinetic limitations. Oral bioavailability of EGCG is extremely low in humans. Research indicates that fewer than 1% of intact EGCG is eliminated via urinary excretion without biotransformation (51). EGCG is rapidly absorbed in the gastrointestinal tract. Peak plasma concentrations are reached within 1–2.5 h. However, its therapeutic efficacy is limited by a short half-life (51). For example, Chow et al. reported that the maximum plasma concentration of EGCG in humans was 77.9 ± 22.2 ng/mL (52). The corresponding AUC was 508.2 ± 227 ng⋅h/mL. The elimination half-life was 3.4 ± 0.3 h (52). These properties collectively restrict therapeutic efficacy.

Finally, dose-dependent hepatotoxicity and narrow therapeutic window. A critical safety concern is that high-dose EGCG can induce dose-related hepatotoxicity (53). The underlying mechanisms are multifactorial. They involve mitochondrial dysfunction and pro-oxidant generation of reactive oxygen species. They also involve topoisomerase II inhibition, which leads to DNA damage (53, 54). Furthermore, fasting increases EGCG absorption. However, high-dose consumption under fasting conditions markedly potentiates the risk of liver injury (53, 55). This underscores a narrow therapeutic window between efficacious and toxic exposures.

Notably, current evidence can only define a general safety boundary rather than establish an individualized therapeutic window for patients with liver fibrosis. Our systematic review of 16 clinical trials revealed that the vast majority were epidemiological observational studies, with only a single dose escalation trial failing to complete all predetermined dose levels (56). None of these studies reported COMT/UGT1A4 genotyping data, thereby precluding the establishment of either a uniform or individualized therapeutic window applicable to liver fibrosis. According to the assessment by the Committee on Toxicity (COT), no hepatotoxicity was observed with daily EGCG intakes below 800 mg for up to 12 months, whereas doses exceeding 800 mg/day resulted in significantly elevated liver enzymes compared with controls. Based on this evidence, the European Food Safety Authority (EFSA) established an acceptable daily intake (ADI) of 322 mg/day for EGCG, and the European Union mandated an upper limit of 800 mg/day in dietary supplements in 2023 (53). Because patients with cirrhosis may exhibit altered drug metabolism, their toxicity threshold may be even lower. Furthermore, emerging evidence from the Minnesota Green Tea Trial identified UGT1A4 (rs6755571) A/C and COMT (rs4680) A/G genotypes as significant risk factors for clinically relevant transaminase elevations (57). These findings strongly suggest that COMT/UGT1A4 polymorphisms may modulate individual susceptibility to EGCG hepatotoxicity. However, in the absence of genotype stratification data from cohorts with hepatic fibrosis, their quantitative impact on the therapeutic window remains unassessable. Consequently, a definitive overlapping interval between effective antifibrotic doses and safe doses devoid of hepatotoxicity risk cannot currently be established for this patient population. Future phase II dose optimization trials in patients with liver fibrosis should incorporate the following elements: (i) multiple dose levels to simultaneously evaluate the efficacy and safety profile; (ii) stratified analysis of COMT/UGT1A4 genotypes; and (iii) inclusion of pharmacokinetic and safety endpoints specific to patients with cirrhosis.

Therefore, overcoming these pharmacokinetic limitations is critical for the clinical translation of EGCG. Bioavailability enhancement strategies are essential. Current approaches can be categorized into three principal domains: advanced delivery systems, chemical structural modification, and pharmaceutical adjuvant strategies.

(1) Nanodelivery and bioactive nano-intervention systems

Passive nanocarriers. (i) Passive nanocarriers rely on the intrinsic physicochemical properties of materials to improve EGCG solubility, storage stability, and *in vivo* pharmacokinetic behavior, without depending on ligand–receptor-specific recognition for tissue targeting. Currently, biodegradable poly(lactic-co-glycolic acid) (PLGA) and chitosan are the most widely employed polymeric materials for EGCG encapsulation (58). PLGA nanoparticles possess sustained-release characteristics and demonstrate hepatoprotective effects significantly superior to free EGCG in cellular and animal models; however, existing studies indicate that polymeric and lipid carriers universally suffer from low drug-loading capacities for hydrophobic drugs, representing a critical bottleneck limiting therapeutic efficacy. (ii) Lipid-based nanomaterials represent the only nanodrug system that has entered the clinical investigation stage in the field of liver fibrosis therapy. Liposomes, composed of phospholipid bilayers structurally analogous to biological membranes, exhibit excellent biocompatibility. They can simultaneously enhance EGCG encapsulation efficiency and storage stability, and nano-EGCG liposomal formulations have been reported to improve oral bioavailability and upregulate α-secretase activity *in vivo* (59). (iii) Beyond polymeric and lipid systems, two classes of inorganic/hybrid nanocarriers—oligomerized EGCG (OEGCG) and EGCG–metal–phenolic networks (MPN)—have also been developed. OEGCG, synthesized via intermolecular polymerization of EGCG monomers, exhibits substantially enhanced hydrophobicity and can self-assemble into nanocores for the co-delivery of therapeutic macromolecules (60). MPN hybrid networks can coat drug-loaded nanocores, enabling stimuli-responsive controlled release in the high reactive oxygen species (ROS) microenvironment characteristic of fibrotic lesions (61). Both systems represent passive delivery platforms that solely ameliorate the physicochemical properties of EGCG.

Active targeting modification and natural bionanoformulations. Passive carriers lack cell selectivity and are prone to systemic off-target accumulation. By contrast, active-targeting nanosystems achieve precise lesion enrichment through surface conjugation of specific ligands that recognize characteristic receptors on activated hepatic stellate cells (aHSCs). (i) Ligand-modified artificial nanosystems. Although existing well-established targeting ligands have not yet been directly applied to EGCG nanoformulations, they provide mature paradigms for the targeted engineering of EGCG carriers. For instance, Jiang et al. employed cyclic cRGD peptide-modified PEG–PLGA nanoparticles to target integrins highly expressed on aHSCs, thereby synergistically inhibiting collagen deposition (62). Retinol-modified nanoparticles can leverage the intrinsic vitamin A uptake pathway of HSCs to achieve targeted enrichment (63). Previous studies have confirmed that membrane peptide functionalization can enhance the uptake efficiency of EGCG nanoparticles by target cells (64); future investigations may graft cRGD or retinol ligands onto the surfaces of PLGA, lipid, OEGCG, and other EGCG-compatible carrier platforms to construct precision-targeted systems. (ii) Natural extracellular vesicles (EVs). Natural EVs are categorized into animal-derived and plant-derived (tea-derived) sources. Stem cell-derived EVs can downregulate fibrosis markers such as collagen and α-SMA (65), whereas tea-derived vesicles are naturally enriched with active nucleic acids including miR-44, directly blocking the TGF-β1 pro-fibrotic pathway (17). Such natural nanocarriers possess both superior biocompatibility and inherent targeting advantages, and may serve as future EGCG co-delivery vehicles to achieve synergistic anti-fibrotic effects between polyphenols and nucleic acid bioactives.

It must be emphasized that all EGCG-loaded nanosystems have undergone pharmacodynamic validation exclusively in short-term small-animal models, and three major bottlenecks impeding clinical translation remain largely unaddressed: (i) fabrication processes such as thin-film hydration and vesicle isolation are currently limited to small-batch laboratory synthesis, lacking standardized industrial scale-up protocols and exhibiting poor batch-to-batch consistency; (ii) existing animal administration periods span only 2–8 weeks, with no available data on chronic organ accumulation or hepatorenal toxicity following long-term repeated dosing; and (iii) the preparation of targeting peptides and pharmaceutical-grade polymers entails prohibitively high costs, with no low-cost synthetic routes currently available. Precisely because of these multifaceted obstacles, clinical trial failure rates for liver disease-related nanomedicines remain persistently high. At present, various EGCG nanodelivery systems remain confined to fundamental research, and a substantial gap persists before human clinical application can be realistically achieved.

(2) Chemical structural modification

Peracetylation and prodrug strategies. Esterification of the phenolic hydroxyl groups generates lipophilic prodrugs. A representative example is peracetylated EGCG (pro-EGCG). This modification masks reactive catechol moieties. It thereby protects against premature glucuronidation and oxidation. Consequently, metabolic stability and membrane permeability are improved. Pro-EGCG exhibits enhanced bioavailability compared to native EGCG. It also exerts sustained therapeutic effects through enzyme-mediated deacetylation in target tissues (66). However, such modifications inevitably alter the polyphenolic scaffold. This may partially attenuate intrinsic antioxidant activity.

(3) Pharmaceutical adjuvants and absorption enhancers

Ascorbic acid (vitamin C). Co-administration of ascorbic acid can protect EGCG from oxidative degradation in the gastrointestinal tract (67). It has also been reported to enhance EGCG stability and intestinal absorption (67).

Piperine. This alkaloid is extracted from black pepper. It inhibits UDP-glucuronosyltransferase (UGT)-mediated glucuronidation. It also inhibits intestinal Phase II metabolism of flavonoids. By suppressing the conjugation of EGCG, piperine substantially elevates plasma concentrations. It also increases systemic exposure (AUC) of free EGCG. These actions amplify its pharmacological effects (68).

### 4.3 Strengths and limitations

This study separately retrieved literature from the WoSCC, Scopus, and PubMed databases rather than merging datasets, which helps guarantee comprehensive data coverage and maintains the integrity of citation-based bibliometric analysis. Several limitations should be acknowledged. First, only English-language publications were included, which might introduce potential language bias and overlook relevant non-English studies. Second, author-level analysis was not performed, because homonymous names are common among Chinese researchers, making accurate author disambiguation difficult. Third, as a pure bibliometric macro-mapping study, this work does not quantitatively assess biological effect sizes, judge the internal validity of individual studies, or measure clinical-translational value. Despite these above-mentioned limitations, our conclusions remain reliable and can provide meaningful macroscopic references for future research in this field.

## 5 Conclusion

Based on scientometric findings, this review identifies four research hotspots in the field: multi-target anti-fibrotic mechanisms, gut–liver axis modulation, synergistic interventions with natural products, and bioavailability optimization. Tea bioactives (particularly EGCG) exert potent anti-fibrotic effects by suppressing hepatic stellate cell activation and extracellular matrix deposition through the TGF-β/Smad, Nrf2, NF-κB, AMPK, and MAPK signaling pathways. Despite its promising efficacy and favorable safety profile, EGCG faces significant hurdles to clinical translation, including poor oral bioavailability, chemical instability, and potential dose-dependent hepatotoxicity. To circumvent these limitations, nano-carrier delivery systems, structural modifications, and absorption enhancers are currently under active investigation. Looking ahead, with the rapid evolution of nanomedicine, rigorously designed multicenter randomized controlled trials, and integrative multi-omics approaches, tea-derived bioactives are poised to overcome existing obstacles and establish their clinical value in the prevention and adjuvant therapy of liver fibrosis.

## Data Availability

The original contributions presented in the study are included in the article/supplementary material, further inquiries can be directed to the corresponding author.
